# REPLY TO: Comments on the journal article entitled “Translation and pilot testing of the ReSPECT form into Danish” published in *Resuscitation Plus May 2026*

**DOI:** 10.1016/j.resplu.2026.101388

**Published:** 2026-06-16

**Authors:** Zoe Fritz, Mette Aaby Smith

**Affiliations:** THIS Institute (The Healthcare Improvement Studies Institute), University of Cambridge, United Kingdom; THIS Institute, Strangeways Research Laboratory, 2 Worts’ Causeway, Cambridge CB2 0AH, United Kingdom; Department of Anesthesiology and Intensive Care, Lillebaelt Hospital, Kolding, University Hospital of Southern Denmark, Denmark; Kolding Sygehus, Sygehusvej 24, DK-6000 Kolding, Denmark

We thank Dr Hardy for their thoughtful comments. The 2025 European Resuscitation council’s *‘Ethics in Resuscitation guidelines’*^1^ emphasise that *“Decisions of DNACPR are best made in the broader context of advance care planning”*. In Denmark, DNACPR has mainly been considered on its own. Our pilot study was conducted as a first step to considering whether Denmark should adopt ReSPECT^2^ nationally.

Denmark currently has no national advance care planning (ACP) documents; each region has its own electrical journal system, which registers ceilings of treatment and CPR recommendations differently. They cannot be accessed by physicians from outside the original hospital where were written. There are two patient-initiated approaches which are nationally accessible: Patients can register a “treatment will” online about their preferences for life-sustaining treatment and/or CPR in the event of them being incapacitated,^3^ and since 2025, persons aged 60 or over can register online, without consulting a physician, to refuse CPR in the case of cardiac arrest.^4^

Education on ACP and DNACPR conversations are not part of the curriculum at the Danish universities and differ between specialties. This is in contrast to the UK where discussions and decisions about CPR and ACP are taught and assessed at medical school.^5^ Specific teaching on ReSPECT has been delivered locally in the UK, but is referenced in Advance Life Support teaching and supported by the Resuscitation Council UKs e-learning for health which is available free to all who work in the NHS – others can also access it.^6^ The Danish project hopes to translate and evaluate this tool and others to support Danish clinicians. For this study the lead author conducted individual teaching of all participating physicians, having received teaching herself in the UK.

Dr Harding is correct that the study had limitations: we chose to recruit from ICU and palliative care in order to identify scenarios where patients would have benefited from ReSPECT conversations. Focus groups and surveys are planned to understand the views of patients and clinicians from other specialities and different grades. The ReSPECT process was introduced in the UK in response to problems with DNACPRs.^7^, ^8^, ^9^ It was designed to improve conversations and documentation, developing a shared understanding between patients and clinicians about which treatments were desired and would realistically lead to valued health outcomes.^10^ In order to introduce the ReSPECT process in a new country in a way that meets the needs of patients, their relatives, and clinicians, we need to understand their views of the current system and what impact ReSPECT would have. The pilot we have published is the first step in doing this. We are currently in receipt of a European Resuscitation Council Grant “ReSPECT from Concept to Continent: A Multicentre Gap Analysis of person-centred resuscitation care and treatment planning”. We are evaluating patient and clinician views of the ReSPECT process in Sweden, Denmark and Poland. This includes considering what educational resource and cultural adaptations are needed to integrate ReSPECT across healthcare settings in these countries; we hope this will be of use to others.

## CRediT authorship contribution statement

**Zoe Fritz:** Conceptualization, Writing – original draft, Writing – review & editing. **Mette Aaby Smith:** Conceptualization, Writing – original draft, Writing – review & editing.

## Declaration of competing interest

Dr Fritz is the chair of the ReSPECT subcommittee for the Resuscitation Council UK. Both Drs Fritz and Smith are contributing to the European Resuscitation Council grant on ReSPECT – explicitly referenced in article.
